# *“Disconnected From Everyone and Everything Around Them”*: A Mixed Method Study to Explore the Perspectives of Children With Language Difficulties and Their Caregivers On Mental Health Concerns and Mental Health Support

**DOI:** 10.1111/1460-6984.70237

**Published:** 2026-04-10

**Authors:** Adrienne Wilmot, Mark Boyes, Natalie Kippin, Daniel Van Heerden, Kat Jones, Suze Leitão, Emily Jackson, Kate Tonta, Bronwyn Myers, Elizabeth Hill

**Affiliations:** ^1^ Curtin enAble Institute Curtin University Perth Western Australia Australia; ^2^ Curtin School of Population Health Curtin University Perth Western Australia Australia; ^3^ Curtin School of Allied Health Curtin University Perth Western Australia Australia; ^4^ Centre for Clinical Interventions Northbridge Western Australia Australia; ^5^ Mental Health, Alcohol, Substance Use, and Tobacco Research Unit South African Medical Research Council Cape Town, Tygerberg South Africa

**Keywords:** developmental language disorder (DLD), language difficulties, mental health, service access

## Abstract

**Background:**

Globally, as many as 1 in 8 children experience severe and persistent language difficulties. These children are at heightened risk for mental health difficulties, however, both the ways in which mental health concerns manifest in the context of language difficulties, and the experiences of children with language difficulties and their caregivers in accessing and engaging with formal mental health supports remain under researched.

**Aim:**

The aim of the current study was to explore lived experience perspectives (child and caregiver) on mental health concerns and, mental health support, for children (≤ 18 years) with language difficulties.

**Method:**

A 2‐phased sequential explanatory design was employed, which combined findings from an online international survey of caregivers (*n* = 120) of children with language difficulties with data from semi‐structured interviews with both children (*n* = 4) and caregivers *(n* = 12).

**Results:**

Combined data from the survey and interviews highlighted a high level of caregiver concern about the mental health of children with language difficulties but low levels of access to formal mental health support. Interviews highlighted several reasons for not accessing formal mental health supports including limited availability of mental health care providers; funding and time constraints (i.e., not wanting to over‐schedule their child); mental health providers lacking knowledge about DLD, and perceived or actual barriers of traditional methods of therapy (e.g., one‐on‐one talk therapy) for children with language difficulties.

**Conclusions and Implications:**

The findings of our study support the view that children with language difficulties are at risk of mental health concerns and yet are also under‐served by mental health services. These findings highlight the need for targeted interprofessional training and integrated models of collaborative care between mental health and language specialists to more adequately meet these children's needs. Future research to engage a wider range of child perspectives and the perspectives of clinicians on the facilitators and barriers to engaging children with language difficulties in formal mental health support is needed.

**Key Points:**

- Children and adolescents with language difficulties experience high rates of anxiety, low self‐esteem, and social difficulties, often in response to academic and peer‐related challenges.
- Despite significant mental health concerns, most families report limited access to formal mental health support, citing barriers such as service unavailability, lack of professional understanding of language difficulties, and the unsuitability of traditional talk‐based therapies.
- There is a critical need for interprofessional collaboration between speech‐language pathologists and mental health professionals, alongside targeted training and inclusive therapeutic approaches, to ensure accessible and effective support for this population.

**WHAT THIS PAPER ADDS:**

*What is already known on this subject*

- Globally, as many as 1 in 8 children experience severe and persistent language difficulties. These children are at heightened risk for mental health difficulties.

*What this study adds to existing knowledge*

- Our study provides lived experience perspectives on mental health concerns and mental health support in the context of language difficulties. The findings highlight caregiver concerns about self‐esteem, anxiety, peer difficulties and the inaccessibility of formal mental health support.

*What are the potential or actual clinical implication of this study?*

- Children with language difficulties are at risk of mental health concerns and yet are also under‐served by mental health services. Findings highlight the need for targeted interprofessional training and integrated models of collaborative care between mental health and language specialists to more adequately meet these children's needs.

## 1 Introduction

Globally, approximately 1 in 8 children experience severe and persistent difficulties using and understanding their own language; a figure that equates to approximately 3 children in every average‐sized classroom (Calder et al., 2022; Norbury et al., 2016). When these difficulties occur outside of autism, intellectual disability, or another neurodevelopmental or biomedical condition in which language disorder can also occur, a child can be considered to have (Developmental) Language Disorder (DLD; American Psychiatric Association (APA) 2013). For most children, language development follows a stable, upward trajectory, becoming more complex and nuanced over time, which facilitates engagement and success in social, academic, and vocational environments, and supports emotional and psychological development (Dubois et al. 2020, Ziegenfusz et al. 2022, Beck et al. 2012). For children with DLD, language does not develop at the same rate and/or to the same extent as their peers, and both academic and socioemotional outcomes can be affected (Mcgregor et al. 2023). Indeed, meta‐analyses demonstrate that language difficulties are a risk factor for both internalising (e.g. anxiety) and externalising (e.g., aggression) mental health concerns in childhood (Donolato et al. 2022, Yew and O'Kearney 2013). Importantly, language difficulties do not appear to be directly associated with poor mental health (Donolato et al. 2022, ST Clair et al. 2019, Lindsay and Dockrell 2012) but may be indirectly associated via emotion regulation challenges (Forrest et al. 2020, Van Den Bedem et al. 2020, Samson et al. 2020) or peer problems (Kilpatrick et al. 2019, Forrest et al. 2018). In terms of peer problems, children with language difficulties are susceptible to bullying victimisation (Redmond 2011), and may be perceived as shy and/or withdrawn (Wadman et al. 2008, Durkin and Conti‐Ramsden 2010). In contrast, many children with DLD exhibit strengths in their social skills (Lloyd‐Esenkaya et al. 2020) and pro‐sociality is protective for the mental health of children with DLD (Conti‐Ramsden et al. 2019, Mok et al. 2014).

Complementing the above‐mentioned quantitative research, a burgeoning body of qualitative studies is providing nuance to our understanding of mental health in the context of DLD (see for example, Burnley et al. 2023, Cullen et al. 2024, Hobson and Lee 2023, Orrego et al. 2023, Wilmot et al. 2024). Importantly, there is a trend among these researchers to highlight the strengths *and* challenges of children (and adults) with DLD (see for example, Cullen et al. 2024, Leitão, 2025) and the ways that mental health difficulties may manifest in the context of language difficulties (Burnley et al. 2023). For example, in their qualitative study, Burnley and colleagues (2023) found that anxiety in children with DLD may manifest as intolerance of uncertainty such as in a desire for predictable daily routines. In another example, Leitão and colleagues (2025) described parental perceptions that children's language difficulties lead to emotion dysregulation, manifesting as meltdowns. Given that children with DLD may become adept at masking their difficulties (Hobson and Lee 2023) and may experience barriers to discussing mental health concerns with caregivers (e.g., a paucity of emotion language; Rieffe and Wiefferink 2017) an examination of mental health concerns in the context of childhood language difficulties is particularly important so that caregivers and educators can be alert to signs of socioemotional difficulties and offer timely support.

Overwhelmingly, lived experience accounts suggest that support for children with DLD needs to go beyond language to also consider their socioemotional wellbeing (see for example, Ekstrom 2022). Yet, concerns remain about the language inaccessibility of formal mental health support (i.e., talk therapy) and its inadequacy for meeting the needs of those with speech, language and communication needs (SLCN; Hancock et al. 2023, Hobson et al. 2022). Indeed, the need for accessible mental health services for children with language difficulties has been recognised as an urgent priority by researchers, clinicians, and people with lived experience (Griffiths et al., 2025, Hobson et al. 2022, Hancock et al. 2023, Hill et al. 2025).

To the best of our knowledge, only one prior study has comprehensively examined the perspectives and experiences of caregivers regarding mental health support for children with language difficulties. Hobson and colleagues (2022) surveyed 74 parents of children with SLCN and also interviewed 9 parents of children with DLD (7–17 years) with current mental health concerns. These researchers found that parents perceived mental health supports to be inaccessible and/or inappropriate for their children due to being verbally mediated; a view that has been corroborated by speech pathologists and mental health professionals in one UK‐based study (Hancock et al. 2023). A stated limitation of Hobson and colleagues (2022) study was the absence of children's own perspectives on the topic. When discussing socioemotional experiences, children's perspectives may not align with those of their parents (Jelen et al. 2023) and behaviours which may be concerning to adults (e.g., social withdrawal) may at times serve a protective function for children's wellbeing from their perspective (Cullen et al. 2024).

### 1.1 The Current Study

To design mental health support for children with language difficulties, it is critical to understand more about the way that mental health concerns manifest in the context of language difficulties and the perspectives and experiences of families regarding mental health support. The aim of the current study was to address these two needs; specifically, to replicate and extend the findings of Hobson and colleagues (2022) to explore lived experience perspectives on mental health concerns and mental health support in the context of language difficulties from both child and caregiver perspectives.

## 2 Methods and Materials

### 2.1 Researcher Positionality

The research team was comprised of researchers with backgrounds in psychology (AW, MB, BM, DVH, KT), speech pathology (EH, SL, EJ, NK) and public health (KJ), many of whom have extensive clinical experience and/or lived experience of parenting neurodivergent children and/or those with SLCN.

### 2.2 Study Design and Materials

A 2‐stage explanatory sequential mixed methods approach was employed to address the research aim (Ivankova et al. 2006). Phase one consisted of quantitative and qualitative data collected via an anonymous online international caregiver survey (*n* = 120). This was followed by Phase 2, qualitative data collected from semi‐structured interviews with caregivers (*n* = 12) and children (*n* = 4) with language difficulties. This approach provided the opportunity to capture breadth and depth of experiences from multiple perspectives. The survey (see S.1) was informed by current literature, including previous survey studies of mental health in the context of child language difficulties (e.g., Hobson et al. 2022), and developed collaboratively by members of the research team. The survey was piloted with three speech pathologists who were not part of the research team, one of whom is the parent of a child with language disorder, and two additional parents of children with DLD. Based on feedback from this pilot, small adjustments to the language of survey items and the survey structure were made to improve clarity. Given the variability in terminology used to refer to language disorder across research, clinical, and community contexts, the survey adopted the term “speech, language, and/or communication difficulties” in individual items as an inclusive descriptor. The interview guides (see S.2 for caregiver and child versions) were developed by experienced qualitative researchers on the research team (BM, SL, AW, LH) and piloted with two parents of children with DLD and a speech pathologist with experience supporting children with DLD. No changes to the interview guides were made.

### 2.3 Phase 1 Procedure

Ethics approval for the study was granted in 2022 by Curtin University Human Research Ethics Commitee. Data collection for Phase 1 (the survey) took place in 2022. Recruitment for the survey occurred via social media, word of mouth, and the professional networks of members of the research team, many of which were DLD‐specific (e.g., The DLD Project). Inclusion criteria for parents/caregivers for the online survey were that participants identified as a parent/caregiver of a “young person” with language disorder. The age criterion for children with language disorder was deliberately broad to capture a range of experiences. Informed consent was collected electronically before participants could proceed to the survey items. Participants were required to provide information about their child's age and the nature of their child's language difficulties within the first section of the survey before providing responses to questions about their child's mental health and perspectives and experience with formal mental health support. The survey took approximately 20 min to complete and could be completed at a time and location of the participant's choosing. Survey participants were not reimbursed for their time.

### 2.4 Phase 1 Participants

A total of 120 caregivers completed the survey. A summary of participant information is provided in table 1 (below). All bar one of the participants answered “Yes” to the screening question: “Does your child experience speech, language or communication difficulties?”. However, this participant was retained in the dataset as they specified that their child had a diagnosis of DLD later in the survey. In terms of diagnoses, the majority (88.4%) of the sample reported that their child had a diagnosis of DLD. However, five caregivers (4%) provided descriptions of their child's speech/language difficulty instead of a specific diagnosis and three caregivers (3%) indicated that, while no formal diagnosis had been made, their child was undergoing speech/language assessment at the time of the survey. Before analysis, a decision was made by the research team to maintain data from all participants in the sample.

**TABLE 1:** Participant demographics (caregiver survey, *N* = 120).

Characteristic	*n*	%
**Country of residence**		
Australia	77	64.2
United Kingdom	32	26.7
Europe	4	3.3
United States	4	3.3
South America	1	0.8
New Zealand	1	0.8
Not reported	1	0.8
**Diagnosis of child**		
DLD (Developmental Language Disorder)	89	74.2
With co‐morbid diagnosis	17	14.2
Language delay	6	5.0
Speech delay	3	2.5
Global developmental delay	2	1.7
Autism spectrum disorder	1	0.8
Apraxia of speech	1	0.8
Speech disorder	1	0.8
Descriptive SLCN only	5	4.2
Undergoing assessment	3	2.5
Not specified	5	4.2
**Speech‐Language Therapy History**		
Currently receiving therapy	71	59.2
Received therapy in the past	47	39.2
Within the past year	10	8.3
Within 1–2 years	23	19.2
More than 2 years ago	7	5.8
Timeframe not specified	7	5.8
Never received therapy	2	1.7

### 2.5 Phase 2 Procedure

The interviews for this study took place between 2022 and 2024. Interview participants were recruited as part of a larger study to engage people with lived experience in the co‐design of a tailored mental health program for children with language difficulties (Jackson et al. 2025). Inclusion criteria were that participants identified as the caregiver of a school‐age child with language disorder (aged ≥ 5 years). Children with language disorder aged ≥ 10 years were also invited to take part. Informed written consent was required prior to participation in the interviews. If children were also participating, then both child assent and parent consent were required. Families could opt for children to be interviewed alone or with a caregiver present. A short online pre‐interview survey was used to gather demographic, diagnostic and mental health information from caregivers. Participants’ responses to the pre‐interview have not been included for analysis. Instead, they were used to inform the interview. All interviews were conducted online via Microsoft Teams by trained/experienced members of the research team (EH, EJ, KJ) using a semi‐structured interview guide (see S.2). Interviews took approximately 45–60 min. All interview participants received a $20 gift voucher to thank them for their participation. Interview participants who reported elevated levels of mental health difficulty in their pre‐interview survey received a follow up call by a psychologist on the team to discuss avenues for support if needed. The audio‐recordings of interviews were transcribed verbatim using artificial intelligence (rev.com) before being de‐identified and checked by hand. Each participant was emailed their transcript to approve before finalising.

### 2.6 Phase 2 Participants

The interview sample consisted of 12 caregivers (11 mothers and 1 father) of children with language difficulties (4–14 years). All children of these caregivers were in mainstream schooling at the time of the interview except for one child who was being homeschooled. Ten of these children had been diagnosed with DLD, one of whom had an additional diagnosis of dyslexia. The other two children had not been diagnosed with DLD but had parent specified language difficulties. One of these children had an autism diagnosis and the other had been diagnosed with autism and speech sound disorder. In addition to the caregivers, the sample consisted of four children (aged 11–14 years) who completed interviews. One child opted to be interviewed alone and the other three opted to be interviewed with their caregiver present.

### 2.7 Analysis

Survey responses were downloaded from Qualtrics into IBM SPSS Statistics (version 29) for analysis. After data cleaning, descriptive statistics (proportions and frequency counts) were calculated. Open‐ended survey responses were analysed using manifest content analysis, following the approach outlined by Bengtsson (2016). This involved identifying and condensing meaning units, assigning descriptive codes to summarise the explicit content of each response, and grouping these codes into categories based on similarities in content. The analysis focused on the surface meaning of caregivers' responses to avoid researcher interpretation (Bengtsson 2016, Graneheim & Lundman, 2004). Qualitative data from the interview transcripts were analysed using reflexive thematic analysis (Braun and Clarke 2022). The survey data and interview data were initially analysed separately by different members of the research team. Then, data from the quantitative and qualitative strands of the study were combined by the lead author in consultation with the last author, under three themes to address the research aim: ***Theme 1: Mental health concerns: Severity and nature***; ***Theme 2: Engagement with formal mental health support*,** and ***Theme 3: The ideal mental health support***. Consistent with an explanatory sequential mixed method design the qualitative data derived from the interviews was used to explain and elaborate on the survey findings. Direct quotations from the interview transcripts were selected to illustrate themes and are included in italics. These quotes have not been corrected for grammar, however, author comments, the removal of irrelevant data and/or combining two sets of data from one participant are all indicated by […] in this manuscript.

## 3 Results

### 3.1 Theme 1: Mental Health Concerns: Severity and Nature

Of 120 caregivers surveyed, 109 (91%) responded ‘yes’ to a question about whether they had ever been concerned about their child's mental health. Of these, 102 (85%) reported they had experienced significant concerns (rating of >3/5) in the past 12 months. When asked about the nature of concerns for their child's mental health (with more than one response allowed), parents selected: self‐esteem/confidence (*n* = 92), social skills/making friends (*n* = 86), anxiety (*n* = 85), bullying victimisation (*n* = 49), anger/aggression (*n* = 44), social withdrawal (*n* = 37), depression (*n* = 30), poor body image (*n* = 19) and bullying perpetration (*n* = 4). Eight caregivers selected ‘other’; their concerns included: emotional regulation/emotional intelligence (*n* = 4), changes at school (*n* = 2), social situations (*n* = 1), impulsivity (*n* = 1), behaviour (*n* = 1), panic attacks (*n* = 1), and anxiety related to literacy skills (*n* = 1).

Consistent with the survey data, caregivers who took part in the interviews raised concerns about their child's mental health. For one child, this had presented in the past as self‐harm and suicidal ideation:

“he's been so anxious and talking about wanting to hurt himself and not wanting to be on this planet anymore and just having massive meltdowns all the time”. One caregiver provided a clear metaphor to illustrate how isolating and anxiety‐inducing the school environment can be for a child with language difficulties, “It's like dropping a kid in ancient Egypt, and him trying to figure out whatever I'm saying and doing and why, and how anxiety riddled that would make anybody, let alone a little kid who doesn't understand what's going on” (P08).

Another caregiver described their child's somatic symptoms of anxiety before school:

“he gets physical symptoms of anxiety like sore stomach, like headache, stress, whatever. And so, he'll complain of, like, feeling sick [….] sometimes I'm like, I don't know if I should keep him home from school or whatever” (P12).

Some caregivers described how their children are prone to internalising negative emotions (e.g., shame or guilt) about school‐related tasks, are extremely sensitive to corrections, and may avoid difficult school tasks. For example, when discussing supporting her child's learning, one caregiver remarked, *ʻʻShe really internalizes it as her fault* […] *even if I'm extremely sensitive to the fact that she's got something wrong, if she feels that she's got something wrong, she completely abandons all progress* [and at another point in the conversation] *“she'll screw it up and she'll put it in the bin*” (P01). One child referred to being “*scared*” at school because their “*teacher was really mean”* and then later in the conversation added *“I didn't get something finished in time and then my teacher got really angry at me when it wasn't even my fault. I just needed extra time”* (C02). Another child told us that “*some teachers still don't understand my DLD*”. The caregiver of this child told us that needing to explain DLD to new people in the school environment was *“causing anxiety”* [for her daughter] *“Just trying to tell new people and not knowing how to. That's her biggest problem, is trying to explain herself”* (P11).

Difficulties with emotion regulation, particularly recognising emotions and regulating frustration were frequently raised by the caregivers interviewed. For example, one caregiver commented that their child's *“ability to check in and identify and act on his own emotions is quite limited”* (P08). Another described the difficulty their child has talking about their emotions: ʻʻ*If you ask him a question, say like, “Do you ever worry about anything, about school or anything, or anything to do with your friends?” He's like, “I don't know.”* (P07). Furthermore, some caregivers discussed how emotion regulation difficulties may impact peer relationships. For instance:

“His regulation is a challenge for him. So, he loves playing with the kids, but he gets really excited and then it's very difficult to bring him down. So that might look like him getting really excited and getting rough and going too far […] or it could be someone doing something he doesn't like and not having the skills to manage that. So might scream and cry. So, it makes him quite vulnerable to bullying” (P04).

Indeed, peer difficulties were concerns that were raised by many participants. One caregiver described feeling “*sad* ” because she perceived her child as being “*very lonely*” [….] *“she's never ever had a friend, never, ever had a friend in her life”* (P10). Across the interviews, relationships with same‐age peers were perceived as particularly challenging. For instance, one child with DLD described feeling “*left out*” socially, especially among people their own age: *“I think around people my age, I get a lot more nervous. I feel like when I'm speaking to an adult, it's a bit less, it's a lot less judgmental”* (C01). In contrast, some caregivers mentioned that their child seeks younger playmates due to their language difficulties, for instance: “*Trying to make friends has been difficult. Just keeping track of what games the kids are playing, talking about what the rules are, yeah, the language that kids his age are using. He tends to play with much younger kids, because the language is obviously simpler”* (P08) and another: “*So the biggest thing I've found is her interaction with kids her age, she'll getalong with kids who are younger”* (P06).

Having close friends seemed to be especially important for children's wellbeing. For instance, one child we interviewed emphasised the importance of friends for a sense of security: “*if it's a lot of people, I'll mostly stick with my friends, I'll feel comfortable, but if I'm away from them I get lots of anxiety”* (C04). Similarly, a caregiver of another child remarked: *“she sticks to a few close friends but she really invests in those friends. So, it's really important to her”* (P01) and another: “*in primary school she used to have to sit next to friends who helped her all the way through primary school”* (P11).

### 3.2 Theme 2: Engagement With Formal Mental Health Support

Despite high levels of parental concern about their child's mental health, 78 of the 120 caregivers surveyed (65%) indicated that their child had never engaged with formal mental health supports. Furthermore, only 15 of the 39 caregivers (38.5%) who had received formal support indicated that the supports were a “good fit” for their child. The formal supports included: clinical psychologists (*n* = 21), school counsellors (*n* = 13), other allied health professionals (e.g., occupational therapists; *n* = 14), mental health workers (*n* = 2), and psychiatrists (*n* = 1). Where ‘other’ was selected (*n* = 3), these included one or more of: GPs (*n* = 2), paediatricians (*n* = 1) and music therapists (*n* = 1). Twenty‐two caregivers (18% of respondents) provided a total of 30 reasons for why a support/service was not a good fit for their child. The most frequent reason was related to therapists’ lack of knowledge/skills about DLD (*n* = 11). This was followed by barriers to accessing timely mental health care (such as costs, lack of services, wait times; (*n* = 5), restricted/limited supports (*n* = 5), lack of impact on the child (*n* = 5), a poor therapeutic relationship (*n* = 2), and lack of parent support and information (*n* = 2). See table 2 below.

**TABLE 2:** Content analysis of responses to “why do you think the formal support was not a good fit for your child's needs?”.

Category	Example
Therapists’ lack of knowledge/skills about DLD	“It was talking therapy which is no good for a child with a language difficulty”
Barriers to accessing timely mental health care (such as costs, lack of services, wait times)	“It is a good fit but cost is prohibitive, and waitlists are impossible”
Restricted/limited supports	“Psychiatrist has been very supportive in his role but it has not been a counselling role therefore our son has not and is not getting support he needs”
Lack of impact on the child	“She finds it very difficult to grasp abstract concepts like feelings so feels like it doesn't have a big impact.”
A poor therapeutic relationship	“The one [service provider] we tried first didn't understand him but pretended he did”
Lack of parent support and information	“A lot more parent support required.”

Consistent with the above‐mentioned survey findings, many of the caregivers who were interviewed discussed barriers to accessing mental health supports/services for their child. These barriers were perceived to relate to (a) poor availability of mental health care services/professionals; (b) funding constraints that limited their access to private providers (c) a lack of professional/specialist knowledge about DLD; and (d) traditional methods of therapy (e.g., one‐on‐one talk therapy) being inappropriate for children with language difficulties due to their high language and communication demands. Furthermore, many caregivers discussed the tension between wanting to provide their child with as much support as possible but not wanting to over‐schedule their child. For instance, when discussing reasons for not pursuing formal mental health support for their child, one caregiver commented, “*I feel like every second day we were in therapy and I'm like poor kid's seeing more office spaces than he does outside”* (P03).

Many caregivers provided their perspectives on difficulties with navigating funding for supports beyond the early years. For instance:

“If the supports were easier to access without getting all these giant waitlists, and not as expensive, because you get cut off… If you are lucky enough to be on NDIS [National Disability Insurance Scheme]^1^, you get your money, but otherwise you just get cut off and that's it” (P07). For one caregiver, who was receiving NDIS funding for her child, the money didn't stretch to psychology:

“At the moment, in our case, if I was to try and involve a psychologist, the NDIS budget, I don't have it. It's been used on speech and OT” (P02).

Additionally, caregivers appeared to be frustrated by a lack of availability, difficulty with timely referrals, and a perception that other neurodevelopmental conditions took priority. For instance: *“it wasn't until year five when I thought, right, we're getting to the pointy end of the stick. And I'd been pushing for help throughout the school. We could not get an appointment with the psychologist, we could not because they were just inundated and pushed to their limits. So, there were kids with autism, ADHD and all that sort of stuff, that obviously took a priority”* (P05). Another caregiver expressed this succinctly, *“Well I guess a massive barrier at the moment is just no one's available*” (P03).

### 3.3 Theme 3: The Ideal Mental Health Support

When asked what would make mental health supports a “better fit” for their child's needs, 30 caregivers (25% of respondents) surveyed provided a total of 34 suggestions. The most frequent suggestion related to having a DLD trained/informed workforce (*n* = 12) followed by improved availability and access to services (*n* = 10). Further suggestions included improved affordability (*n* = 5), access to multi/trans‐disciplinary services (*n* = 3), a better therapeutic relationship between the therapist and child (*n* = 2) and having personalised/person‐centred therapeutic targets (*n* = 2). See table 3 below.

**TABLE 3:** Content analysis of responses to question “what changes could be made to make the formal supports a better fit for your child's needs?”.

Category	Example
Having a DLD trained workforce	“The professional needs to have training in DLD and how to communicate with a child with a language difficulty”
Improved availability and access to services	“Just more appointments available”
Improved affordability	“I can't afford psych input on top of tutoring and SP”
Access to multi/trans‐disciplinary services	“As we understood more, we looked for different perspectives and experiences from therapy team”
A better therapeutic relationship between the therapist and child	“Find a way to engage her in a way she understands and [is] interested in…”
Having personalised/person‐centred therapeutic targets	“It also needs to be more focussed on the daily difficulties caused by DLD.”

Consistent with the survey findings, having mental health professionals with a good understanding of DLD was considered a high priority for the caregivers who were interviewed. For instance, one caregiver remarked: *“I want someone who actually knows DLD … I don't want to have to go through explaining it and have to educate them on it before they look at these concerns”* (P01).

In terms of formal mental health support, many caregivers perceived that traditional methods of therapy (i.e., “talk therapy”) were inaccessible due to language demands. For instance, one caregiver remarked: “*If a therapy is talk‐based, that's where it won't work”* (P04) and another commented: “*we go to these psychology sessions and she'd* [her daughter] *just talk about being fine, everything's fine, “I'm fine.” And she just couldn't articulate even what it felt like in those moments*” (P09). Some believed that one‐one‐one emotional conversations, which rely heavily on language and communication skills may elicit anxiety for children with language difficulties. It seemed that caregivers want interventions to be delivered in a manner that is comfortable and accessible for their children. Caregivers offered suggestions such as movement and art therapy as alternatives to traditional talk‐based therapy. In keeping with this, one child we interviewed described how movement aids their communication and helps them to feel better: *“one reason I do like walking and talking is because when you walk it, like stimulates your brain, um which lets you like, say more and things”* (C03).

Consistent with the responses from our survey, many caregivers discussed the importance of increasing community awareness of DLD to support socioemotional wellbeing. For instance:

“If the understanding about DLD was there, I feel like we wouldn't need as much of the mental health support […] because it's the poor kids are feeling like they are disconnected from everyone and everything around them, because they can't understand what's going on and relate to people and have people relate to them where they are. That makes them feel so isolated and sad and anxious. If the supports were there, and the understanding was there, I don't think you would need as much mental health support” (P08).

## 4 Discussion

The aim of the current study was to explore lived experience (caregiver and child) perspectives on mental health concerns and mental health support in the context of childhood language difficulties. A 2‐phased sequential explanatory design was employed to combine findings from an online anonymous, international survey of 120 caregivers with data from semi‐structured interviews including the perspectives of 12 caregivers and 4 children. Whilst the interview sample were Australian based, survey data were collected from caregivers from across the globe (64% Australian).

Consistent with past research (see for example, Burnley et al. 2023, Lloyd‐Esenkaya et al. 2021, Hobson et al. 2022) the findings of our study highlighted a significant degree of caregiver concern about the mental health of children with language difficulties. Whilst both internalising (e.g., social withdrawal) and externalising (e.g., aggression) symptoms were reported, difficulties with self‐esteem and anxiety were the most reported mental health concerns across the survey and interview responses. Importantly, in our study, anxiety was reported by children themselves as well as their caregivers. Consistent with caregiver perspectives gathered in previous studies (see for example, Leitão et al., 2025) our study highlighted that poor self‐esteem and anxiety among children with language difficulties is often in response to the academic and/or social demands of school. Caregivers discussed anxiety manifesting as: somatic symptoms before school, avoidance of homework/schoolwork, and meltdowns. Children reported experiencing pressure at school and some had felt misunderstood by teachers. Findings such as these highlight the importance of teacher education about the associations between language, literacy and mental health, and the ways that anxiety can manifest in children with language difficulties. Our results showed that caregivers perceived that school staff/teachers often lacked knowledge about/skills to support DLD and co‐occurring mental health difficulties. This finding is consistent with previous research (Ramsay et al. 2018, Ciullo & Hoover, 2025, Glasby et al. 2022) and highlights the need for a DLD informed workforce more broadly across both schools and formal mental health support services. Difficulties with self‐esteem present a significant risk for future mental health difficulties including the development of depression, anxiety, eating disorders, and difficulties with social relationships and academic and occupational achievement (Fairburn et al. 2003, Orth and Robins 2022) and should therefore be identified and addressed as early as possible in a child's development.

In addition to self‐esteem and anxiety, caregivers expressed concerns about their child's social difficulties. Specifically, difficulties with bullying victimisation, social skills/making friends, and social withdrawal/loneliness were reported. This finding is concerning given strong links between these social difficulties and internalising symptoms among children with and without language difficulties (Kwan et al. 2020, Kilpatrick et al. 2019, Achterbergh et al. 2020). Importantly, and in keeping with recent literature (for example, Lloyd‐Esenkaya et al. 2020) caregivers highlighted both the peer difficulties *and* strengths of their children. Whilst many children had experienced bullying due to their language difficulties and some reported loneliness, others had developed strategies to overcome peer difficulties such as opting to play/socialise with younger children and investing in quality friendships. Overwhelmingly, consistent with past research (see for example, Cullen et al. 2024) the children in our sample were socially motivated. Indeed, there was a sense that friends may have particular significance to children with DLD. For example, one of the children we interviewed commented that the emotional support provided by friends at school helped mitigate their anxiety. Implications of these findings are the importance of early intervention to promote social skills in children with language difficulties, whole school interventions to foster understanding and inclusion, and teacher strategies to promote friend support in school settings (e.g., allowing friends to work together). Links between language, emotion regulation (both awareness and regulation), and mental health were highlighted by the caregivers interviewed, in keeping with past literature (see for example, Forrest et al. 2020). Future research, building on preliminary work by Durgungoz & ST Clair (2024) is needed to investigate how emotion regulation can be effectively taught to children with DLD and if this benefits their mental health.

Despite high levels of mental health support needs, most caregivers surveyed in our study had never accessed formal mental health supports for their child, consistent with findings from Hobson and colleagues (2022). Reasons for not accessing formal mental health supports included (a) a poor availability of mental health care services/professionals; (b) funding and time (e.g., not wanting to over‐schedule their child) constraints (c) a lack of professional/specialist knowledge about DLD; and (d) the perceived or actual inaccessibility of traditional methods of therapy (e.g., one‐on‐one talk therapy) for children with language difficulties. Concerns about limited service availability in our sample echo broader issues identified within contemporary child mental health literature (Subotic‐Kerry et al., 2025). However, it is possible that the experience(s) of service unavailability is exacerbated for children with language difficulties and their caregivers, who described the process of repeatedly encountering, or anticipating, reduced understanding of DLD (and its impact on mental health) as an exhausting experience. This emotional burden, particularly among caregivers, appeared to stem from their need to persistently advocate for their child's needs and from their sense of responsibility for ‘educating’ professionals about DLD when navigating pathways to service access. Parents’ frustration highlights a broader and systemic issue related to the perceived lack of professional competence to support children with co‐occurring mental health and language difficulties (Hill et al. 2024) and the unsuitability of “talking therapies” which are the common practice used with children—even those experiencing language difficulties (Hill et al. 2025). When a mental health practitioner lacks a foundational understanding of DLD (and SLCN more broadly), it may undermine their ability to identify and appropriately support children with these challenges and their caregivers (Hancock et al. 2023, Hill et al. 2024). These findings highlight the need for targeted interprofessional training and models of collaborative care between mental health and speech‐language pathologists to adequately meet these children's needs (Griffiths et al., 2025, Hancock et al. 2023, Hobson et al. 2022).

The mixed methods design of our study was a strength which facilitated the breadth and depth of our analysis. However, we were only able to recruit 4 children to our study and the absence of clinician/educator perspectives is a further limitation. As a result, child perspectives were not reflected across all themes and future research should consider the perspectives of clinicians/educators. Furthermore, it is plausible that caregivers who were concerned about their children's mental health were more likely to volunteer, potentially leading to an over‐estimation of levels of caregiver concern that may not reflect those among the broader population of families of children with language difficulties. Future research is needed to further investigate the perspectives of clinicians on barriers and enablers to providing timely, affordable and accessible mental health support for children with language difficulties, and importantly, the perspectives of a wider range of children.

## 5 Conclusion

In conclusion, our findings highlighted a high level of caregiver concern for child mental health, particularly in terms of anxiety, self‐esteem, and peer relationships. Despite this level of concern, access to formal mental health supports was low, and caregivers perceived multiple barriers to their child receiving formal mental health support that suited their language needs. Our findings therefore replicate and extend those of Hobson and colleagues (2022) to highlight children with language difficulties as a population that is at risk of experiencing mental health concerns yet are under‐served by our mental health system. Future research should work with stakeholders to co‐design accessible mental health programs to support the needs of children with language difficulties and their families.

## Funding statement

This work was supported by the Curtin enAble Institute, The DLD Project (International DLD Research Grant), and Healthway (grant number 34838). Adrienne Wilmot was supported by Mark Boyes' National Health and Medical Research Council (NHMRC), Australia (Investigator Grant 1173043) and the Medical Research Future Fund (MRF2025822). Mark Boyes was supported by the NHMRC, Australia (Investigator Grant 1173043) and the Stan Perron Charitable Foundation (People Grant 202405). Bronwyn Myers was supported by a Stan Perron Charitable Foundation People Grant (230526). Elizabeth Hill was supported by a Stan Perron Charitable Foundation People Grant (202552).

## Ethical approval and informed consent statements

This study was approved by the Curtin University Human Research Ethics Committee (HRE2022‐0383). Informed consent was obtained from all participants before data collection. For child participants, informed consent was obtained from parents/caregivers, and age‐appropriate assent was obtained from children prior to participation. Participants were assured of the confidentiality of their responses and their right to withdraw from the study at any time without consequence.

## Conflicts of Interest

The author(s) declared no potential conflicts of interest with respect to the research, authorship, and/or publication of this article.

## Supporting information




**Supplementary Material**: S.1 survey S.2 pre‐interview survey S.3 interview guide

## Data Availability

The data that support the findings of this study are available from the corresponding author (EH), upon reasonable request.

## Endnotes

1 The NDIS funds Australians (aged 9‐65 years) with disability to access the services and supports they need. To meet inclusion criteria the disability must be considered to be caused by a “permanent impairment” https://www.ndis.gov.au/.
