# Supplementary material for: “Disconnected From Everyone and Everything Around Them”: A Mixed Method Study to Explore the Perspectives of Children With Language Difficulties and Their Caregivers On Mental Health Concerns and Mental Health Support
Source: Int J Lang Commun Disord. 2026 Apr 10;61(3):e70237. doi: 10.1111/1460-6984.70237 (PMC13067927; doi:10.1111/1460-6984.70237)
Supplement: Supplementary file 1 — Supplementary Material: S.1 survey S.2 pre‐interview survey S.3 interview guide [file JLCD-61-0-s001.docx]

S1 Caregiver survey (hosted via Qualtrics)

*Note:* Skip logic has been retained.

This section will start with a few questions about **you and your child**. No identifying information will be collected. Please tell us the country in which you live

________________________________________________________________

Are you a parent or caregiver to a young person (<18 years)?

- Yes
- No

Skip To: End of Survey If Are you a parent or caregiver to a young person ( 18 years)? = No

How old is your child in **years and months** (e.g., 12 years 3 months)?

________________________________________________________________

Does your child experience **speech, language, or communication difficulties?**

- Yes
- No
- Unsure

Does your child have a **diagnosed speech, language, or communication difficulty or disorder**?

- Yes. Please describe __________________________________________________
- No
- Unsure

Has your child ever seen a **speech pathologist** (/speech-language therapist)?

- Yes, currently
- Yes, in the past. Please tell us how long ago (e.g., 1 year) __________________________________________________
- No
- Unsure

We are interested in your experience(s) accessing and engaging with support for your child's emotional wellbeing and mental health. **Emotional wellbeing** refers to our ability manage our emotions and adapt to change and difficult times. **Mental health** refers to our emotional wellbeing, as well as psychological and social wellbeing, and how we think, feel, and act. The next questions will ask about your child's emotional wellbeing and mental health, as well as their experiences with any supports or services for their wellbeing.
 

Have you **ever** been concerned about your child's emotional wellbeing or mental health?

- Yes
- No
- Unsure

Display this question:

If Have you ever been concerned about your child's emotional wellbeing or mental health? = Yes

Or Have you ever been concerned about your child's emotional wellbeing or mental health? = Unsure

**Please rate your level of concern** about your child's emotional wellbeing (1, not at all concerned, 5 very concerned)?

|  | 1 (not at all) | 2 | 3 | 4 | 5 (very concerned) |
| --- | --- | --- | --- | --- | --- |
| In the past 12 months |  |  |  |  |  |
| Right now |  |  |  |  |  |

Display this question:

If Have you ever been concerned about your child's emotional wellbeing or mental health? = Yes

Or Have you ever been concerned about your child's emotional wellbeing or mental health? = Unsure

**What are, or were, your concerns** about your child's emotional wellbeing or mental health? Please select all that apply.

- Self-esteem / confidence
- Being bullied
- Bullying others
- Depression
- Anxiety
- Anger / aggression
- Social skills / making friends
- Withdrawal
- Substance abuse
- Body image
- Other (please describe) __________________________________________________

Has your child ever received **formal** **support** for their emotional wellbeing? **Formal support** refers to services provided by a psychologist or psychiatrist as well as school-based counselling, or supports provided by other health services.

- Yes
- No
- Unsure

Display this question:

If Has your child ever received formal support for their emotional wellbeing?Formal support refers t... = Yes

Please tell us **what prompted you to seek formal support** for your child's emotional wellbeing or mental health (e.g., referral, your concern, teacher concern).

________________________________________________________________

Display this question:

If Has your child ever received formal support for their emotional wellbeing?Formal support refers t... = Yes

Or Has your child ever received formal support for their emotional wellbeing?Formal support refers t... = Unsure

Approximately **how long after a concern was identified did you seek formal support** for your child's emotional wellbeing or mental health? Please state

________________________________________________________________

Display this question:

If If Approximately how long after a concern was identified did you seek formal support for your child's emotional wellbeing or mental health? Please state Text Response Is Displayed

Approximately **how long after contacting a service provider** (e.g., psychology clinic, or school-based service) **did your child begin receiving formal support** for their emotional wellbeing or mental health? Please state

Display this question:

If Has your child ever received formal support for their emotional wellbeing?Formal support refers t... = Yes

Or Has your child ever received formal support for their emotional wellbeing?Formal support refers t... = Unsure

Did your child receive a **specific program** or **specific approach** (e.g., Cognitive Behavioural Therapy [CBT]) designed to support emotional wellbeing or mental health?

- Yes. Please describe. __________________________________________________
- No
- Unsure

Display this question:

If Has your child ever received formal support for their emotional wellbeing?Formal support refers t... = Yes

Or Has your child ever received formal support for their emotional wellbeing?Formal support refers t... = Unsure

What was, or were, the **target**(s) of the formal support your child received for their emotional wellbeing or mental health? Please select all that apply

- Self-esteem / confidence
- Being bullied
- Bullying others
- Depression
- Anxiety
- Anger / aggression
- Social skills / making friends
- Withdrawal
- Substance abuse
- Body image
- Other (please describe)
- I don't know

Display this question:

If Has your child ever received formal support for their emotional wellbeing?Formal support refers t... = Yes

Or Has your child ever received formal support for their emotional wellbeing?Formal support refers t... = Unsure

**How did you find out** about the formal support that was available for your child for their emotional wellbeing or mental health? Please select all that apply.

- GP
- School counsellor
- School teacher
- Allied health (e.g., speech pathologist or occupational therapist)
- On the internet
- Word-of-mouth (family or friends)
- Other. Please describe. __________________________________________________
- I don't know

Display this question:

If How did you find out about the formal support that was available for your child for their emotion... , GP Is Displayed

**Where** did the formal support for your child's emotional wellbeing or mental health take place? Please select all that apply.

- Home
- Online
- School
- Clinic (e.g., psychology clinic)
- Other location(s) / mode. Please describe. __________________________________________________

Display this question:

If Has your child ever received formal support for their emotional wellbeing?Formal support refers t... = Yes

Or Has your child ever received formal support for their emotional wellbeing?Formal support refers t... = Unsure

**Who** provided the formal support for your child's emotional wellbeing or mental health to your child? Please select all that apply.

- Clinical psychologist
- Psychiatrist
- School counsellor
- Mental health worker
- Allied health (e.g., speech pathologist or occupational therapist)
- Other. Please describe,
- I don't know

Display this question:

If Has your child ever received formal support for their emotional wellbeing?Formal support refers t... = Yes

Or Has your child ever received formal support for their emotional wellbeing?Formal support refers t... = Unsure

These questions will ask for your opinions on the formal support your child received for their emotional wellbeing

Display this question:

If Has your child ever received formal support for their emotional wellbeing?Formal support refers t... = Yes

Or Has your child ever received formal support for their emotional wellbeing?Formal support refers t... = Unsure

Do you think the formal support your child received a **was a good fit** for their needs? You may refer to **needs related to emotional wellbeing and mental health** as well as **language and communication needs**.

- Yes
- No
- Unsure

Display this question:

If Do you think the formal support your child received a was a good fit for their needs? You may ref... = Yes

Or Do you think the formal support your child received a was a good fit for their needs? You may ref... = Unsure

Why do you think the formal support **was a good fit** for your child's needs? You may refer to **needs related to emotional wellbeing and mental health** as well as **language and communication needs**.

Display this question:

If Do you think the formal support your child received a was a good fit for their needs? You may ref... = No

Or Do you think the formal support your child received a was a good fit for their needs? You may ref... = Yes

Or Do you think the formal support your child received a was a good fit for their needs? You may ref... = Unsure

Why do you think the formal support **was not a good fit** for your child's needs? You may refer to **needs related to emotional wellbeing and mental health** as well as **language and communication needs**.

Display this question:

If Do you think the formal support your child received a was a good fit for their needs? You may ref... = Yes

Or Do you think the formal support your child received a was a good fit for their needs? You may ref... = No

Or Do you think the formal support your child received a was a good fit for their needs? You may ref... = Unsure

What changes could be made to make the formal support a **better fit** for your child's needs? You may refer to **needs related to emotional wellbeing and mental health** as well as **language and communication needs**.

Display this question:

If Has your child ever received formal support for their emotional wellbeing?Formal support refers t... = No

These questions will ask for your opinions on formal support for your child's emotional wellbeing

Display this question:

If Has your child ever received formal support for their emotional wellbeing?Formal support refers t... = No

If your child was to receive support for their emotional wellbeing or mental health, **what would be your priorities**? (e.g., anxiety, self-esteem, confidence)

Display this question:

If Has your child ever received formal support for their emotional wellbeing?Formal support refers t... = No

If your child was to receive support for their emotional wellbeing or mental health, what **mode of delivery** would be best? Select all that apply.

- Face-to-face (e.g., at a clinic)
- School-based program
- Home-based program
- Online program
- Parent-led
- Combined support with other allied health (e.g., speech pathology or occupational therapy)
- Other. Please describe.
- I don't know

Display this question:

If Has your child ever received formal support for their emotional wellbeing?Formal support refers t... = No

What kind of support do **you think** would be a **good fit** for your child's needs? You may refer to **needs related to emotional wellbeing** **and mental health** as well as **language and communication needs.**

Display this question:

If Has your child ever received formal support for their emotional wellbeing?Formal support refers t... = No

What kind of support do **you think would not be a good fit** for your child's needs? You may refer to **needs related to emotional wellbeing and mental health** as well as **language and communication needs.**

Is there anything else you would like to tell us about your child's experience(s) with accessing mental health support ?

- No, thank you
- Yes __________________________________________________

**S2 Pre-interview Survey (Parent/caregiver)**

Please tell us your name (first and last):

________________________________________________________________

Please tell us your child's name (first and last)

________________________________________________________________

My child is:

Younger than 10 years old

Older than 10 years old

(Note: The purpose of this question was to determine whether not the child would be eligible to register their interest in an interview).

How old is your child in years and months (e.g., 10 years and 2 months)?

________________________________________________________________

Does your child have a diagnosed language disorder or difficulty?

Yes. Please specify (e.g., Developmental Language Disorder) __________________________________________________

No. Please describe the language difficulties they experience __________________________________________________

Unsure. Please describe any language difficulties they experience __________________________________________________

Is your child aware of their diagnosis? This is important, as it will help us know how to best refer to your child's language difficulties, or disorder, during our conversations and workshops. Please type your answer, below.

________________________________________________________________

Have you ever been concerned about your child's mental health?

Yes

No

Unsure

Please rate your level of concern about your child's mental health or emotional wellbeing in the past 12 months (1, not at all concerned, 5 very concerned):

Please rate your level of concern about your child's mental health or emotional wellbeing right now (1, not at all concerned, 5 very concerned):

Please describe the nature of your concerns for your child's mental health or emotional wellbeing (currently or in the past):

________________________________________________________________

Has your child ever received formal support for their mental health or emotional wellbeing? Formal support refers to services provided by a psychologist or psychiatrist as well as school-based counselling, or supports provided by other health services.

Yes, currently

Yes, in the past. Please specify how long ago

No

Please tell us who provided this formal support to your child (e.g., psychiatrist, psychologist, school counsellor):

What were the priorities of the formal support your child received for their mental health or emotional wellbeing (e.g., behaviour, anxiety, depression)?

________________________________________________________________

Please rate how effective the formal support was for helping your child's mental health or emotional wellbeing (0 = not at all effective, 5 = very effective):

Were there any barriers to your child receiving formal support for their mental health or emotional wellbeing (e.g., travel, referral, time)? Please describe:

What helped your child receive formal support for their mental health or emotional wellbeing? Please describe:

The next questions will ask about your (parent/caregiver) thoughts and otions. In the past four (4) weeks....

|  |  |  |  |  |  |
| --- | --- | --- | --- | --- | --- |
|  | None of the time | A little of the time | Some of the time | Most of the time | All of the time |
| How often did you feel tired out for no good reason? |  |  |  |  |  |
| How often did you feel nervous? |  |  |  |  |  |
| How often did you feel so nervous that nothing could calm you down? |  |  |  |  |  |
| How often did you feel hopeless? |  |  |  |  |  |
| How often did you feel restless or fidgety? |  |  |  |  |  |
| How often did you feel so restless you could not sit still? |  |  |  |  |  |
| How often did you feel depressed? |  |  |  |  |  |
| How often did you feel that everything was an effort? |  |  |  |  |  |
| How often did you feel so sad that nothing could cheer you up? |  |  |  |  |  |
| How often did you feel worthless? |  |  |  |  |  |

These questions will ask you (child/young person) about the way you think and feel.  If you ave difficulty understanding the next questions, your parent can read the items out loud to you. Parents, please do not influence your child's answers in any way.

|  | Not True | Somewhat True | Certainly True |
| --- | --- | --- | --- |
| I try to be nice to other people. I care about their feelings |  |  |  |
| I am restless, I cannot stay still for long |  |  |  |
| I get a lot of headaches, stomach-aches or sickness |  |  |  |
| I usually share with others, for example CD’s, games, food |  |  |  |
| I get very angry and often lose my temper |  |  |  |
| I would rather be alone than with people of my age |  |  |  |
| I usually do as I am told |  |  |  |
| I worry a lot |  |  |  |
| I am helpful if someone is hurt, upset or feeling ill |  |  |  |
| I am constantly fidgeting or squirming |  |  |  |
| I have one good friend or more |  |  |  |
| I fight a lot. I can make other people do what I want |  |  |  |
| I am often unhappy, depressed or tearful |  |  |  |
| Other people my age generally like me |  |  |  |
| I am easily distracted, I find it difficult to concentrate |  |  |  |
| I am nervous in new situations. I easily lose confidence |  |  |  |
| I am kind to younger children |  |  |  |
| I am often accused of lying or cheating |  |  |  |
| Other children or young people pick on me or bully me |  |  |  |
| I often volunteer to help others (parents, teachers, children) |  |  |  |
| I think before I do things |  |  |  |
| I take things that are not mine from home, school or elsewhere |  |  |  |
| I get along better with adults than with people my own age |  |  |  |
| I have many fears, I am easily scared |  |  |  |
| I finish the work I'm doing. My attention is good |  |  |  |

These questions will ask you (parent/caregiver) about the way your child thinks and feels. Please indicate how true each of these statements is about your child.

|  | Not True | Somewhat True | Certainly True |
| --- | --- | --- | --- |
| Considerate of other people's feelings |  |  |  |
| Restless, overactive, cannot stay still for long |  |  |  |
| Often complains of headaches, stomach-aches or sickness |  |  |  |
| Shares readily with other children, for example toys, treats, pencils |  |  |  |
| Often loses temper |  |  |  |
| Rather solitary, prefers to play alone |  |  |  |
| Generally well behaved, usually does what adults request |  |  |  |
| Many worries or often seems worried |  |  |  |
| Helpful if someone is hurt, upset or feeling ill |  |  |  |
| Constantly fidgeting or squirming |  |  |  |
| Has at least one good friend |  |  |  |
| Often fights with other children or bullies them |  |  |  |
| Often unhappy, depressed or tearful |  |  |  |
| Generally liked by other children |  |  |  |
| Easily distracted, concentration wanders |  |  |  |
| Nervous or clingy in new situations, easily loses confidence |  |  |  |
| Kind to younger children |  |  |  |
| Often lies or cheats |  |  |  |
| Picked on or bullied by other children |  |  |  |
| Often volunteers to help others (parents, teachers, other children) |  |  |  |
| Thinks things out before acting |  |  |  |
| Steals from home, school or elsewhere |  |  |  |
| Gets along better with adults than with other children |  |  |  |
| Many fears, easily scared |  |  |  |
| Good attention span, sees work through to the end |  |  |  |

**S3 Semi-structured Interview Protocol (Parent)**

*Introductory pre-amble.*

Do you have any questions for me before we start?

1. Firstly, what motivated you to work with us on this project?
2. In the online survey, you told us that **CHILD’S NAME** has a diagnosis of DLD

Could you tell me a bit about their diagnosis and what this has meant for them?

*Prompt:* what about any impact of the diagnosis on day to day life?

1. In the online survey, you told us that **CHILD’S NAME** has difficulties with…

Could you tell me a bit about these difficulties and what this has meant for CHILD NAME?

*Prompt:* what about any impact of language difficulties on day to day life?

1. Does your child receive speech pathology or other support for their language and communication?

If yes, can you tell me more about it?

If no, has anything influenced their access to speech pathology services or other support?

1. You mentioned that you are / have been **SPECIFY LEVEL OF CONCERN** about **CHILD’S NAME’s** emotional wellbeing. Could you tell me a bit more about why you gave this rating?
2. What factors have you noticed that have a positive impact on **CHILD NAME’s** emotional wellbeing?
3. What factors have you noticed that have a negative impact on **CHILD NAME’s** emotional wellbeing?
4. In the survey, you told us that **CHILD’S NAME** has received some support for their emotional wellbeing. I’m keen to hear a bit more about that, would you feel comfortable sharing some more information with me?
   1. Describe the kind of support, e.g., was it a formal program like Aussie Optimism
   2. Who referred CHILD NAME? How did you find out about support that was available?
   3. How long did you wait for help?
5. IF NOT ALREADY COVERED - You listed **LIST PRIORITIES FROM SURVEY** as the main priorities for this support. Could you describe **CHILD NAME’s** experiences with these areas a bit more?
6. In terms of the success or effectiveness of this support for **CHILD NAME,** you rated this as a **PROVIDE RATING / 5.**

What made this support successful?

Why do you think this support was not effective for **CHILD NAME**?

Is there anything more you’d like to share about you, or your child’s, experience with their emotional wellbeing or support services? This information is really helpful for guiding our workshops. Do you have any questions for me?
